# Speech Facilitation by Left Inferior Frontal Cortex Stimulation

**DOI:** 10.1016/j.cub.2011.07.021

**Published:** 2011-08-23

**Authors:** Rachel Holland, Alex P. Leff, Oliver Josephs, Joseph M. Galea, Mahalekshmi Desikan, Cathy J. Price, John C. Rothwell, Jennifer Crinion

**Affiliations:** 1 Institute of Cognitive Neuroscience, University College London, 17 Queen Square, London WC1N 3AR, UK; 2 Wellcome Trust Centre for Neuroimaging, University College London, 12 Queen Square, London WC1N 3BG, UK; 3 Birkbeck-UCL Centre for Neuroimaging, 26 Bedford Way, London WC1H 0AP, UK; 4 Human Movement and Balance Unit, Institute of Neurology, 33 Queen Square, London WC1N 3BG, UK

## Abstract

Electrophysiological studies in humans and animals suggest that noninvasive neurostimulation methods such as transcranial direct current stimulation (tDCS) can elicit long-lasting [1], polarity-dependent [2] changes in neocortical excitability. Application of tDCS can have significant and selective behavioral consequences that are associated with the cortical location of the stimulation electrodes and the task engaged during stimulation [3–8]. However, the mechanism by which tDCS affects human behavior is unclear. Recently, functional magnetic resonance imaging (fMRI) has been used to determine the spatial topography of tDCS effects [9–13], but no behavioral data were collected during stimulation. The present study is unique in this regard, in that both neural and behavioral responses were recorded using a novel combination of left frontal anodal tDCS during an overt picture-naming fMRI study. We found that tDCS had significant behavioral and regionally specific neural facilitation effects. Furthermore, faster naming responses correlated with decreased blood oxygen level-dependent (BOLD) signal in Broca's area. Our data support the importance of Broca's area within the normal naming network and as such indicate that Broca's area may be a suitable candidate site for tDCS in neurorehabilitation of anomic patients, whose brain damage spares this region.

## Highlights

► This is a novel application of concurrent A-tDCS and fMRI during speech production ► Left frontal A-tDCS speeds up spoken naming responses ► Left frontal A-tDCS elicits a regionally specific neural effect in Broca's area ► Decreased BOLD signal in Broca's area correlates with faster naming responses

## Results and Discussion

Recent research suggests a relationship between primarily left inferior frontal cortex (IFC) activity, including Broca's area, and improved naming performance in stroke patients with aphasia [14–17]. Moreover, anodal transcranial direct current stimulation (A-tDCS), a method thought to increase cortical excitability, over Broca's area has been shown to enhance naming accuracy in aphasic patients [6]. As such, A-tDCS has been proposed as a clinical tool for rehabilitation of specific language function in brain-damaged patients and as a supplementary treatment approach for anomia [15]. In healthy speakers, A-tDCS of Broca's area has resulted in (1) an improved rate of verbal fluency [18] and (2) facilitation of language skills when paired with a learning paradigm [3, 4]. Surprisingly, there have been no studies investigating whether A-tDCS applied to this region can be used to facilitate naming. Here we tested whether A-tDCS over the left IFC can be used to increase spoken picture-naming performance in neurologically unimpaired individuals.

Behavioral priming (BP) is a term used to describe an improvement in performance as a result of repeated encounters with the same or related stimuli. For example, in the context of a naming task, faster response latencies can be achieved in healthy speakers by repeated encounters with the same picture stimuli, i.e., the response to a second presentation of a stimulus will be faster than the first (an order effect), or crossmodal repetition of the object's name, e.g., naming a picture of a car while concurrently hearing “car” will be faster than naming “car” without a related auditory cue [19]. Neural priming (NP) is a term used to describe a reduction in task-dependent neural activity that typically accompanies BP [20]. In functional magnetic resonance imaging (fMRI) studies, the signature of NP is a reduction of blood oxygen level-dependent (BOLD) signal. The modification of synaptic thresholds is fundamental to neuronal adaptation paradigms. Likewise, A-tDCS—by depolarizing neurons nearer to threshold—can reduce the amount of excitatory input required to produce a naming response. Thus, we can have a situation in which there is increased excitability (manifest as a faster response time to a given input) accompanied by reduced BOLD (less synaptic input for a given output, in this case naming). Similar results have been observed by Antal and colleagues [12] where anodal stimulation of motor cortex reduced supplementary motor area (SMA) activation during a finger-thumb motor skill task. However, the opposite result was reported by Kwon and Jang [11], who found increased activation in the motor cortex during a hand grip task with anodal stimulation. An important difference between these studies and the study reported here is that the previous studies were not assessing a change in behavior: performance was maintained during sham and A-tDCS conditions. Consequently, brain activity changes associated with a behavioral improvement resulting from tDCS were not measured. Our aim was to determine whether A-tDCS applied to the left IFC has a facilitatory effect on naming, a focal effect in Broca's area, and whether anodal stimulation interacts with established order and crossmodal repetition BP effects. Our hypothesis was that A-tDCS applied to left IFC would result in (1) an independent facilitation effect on naming, i.e., faster naming responses, with (2) an accompanying regionally specific reduced BOLD response in Broca's area, analogous to NP effects.

**Figure 1:**
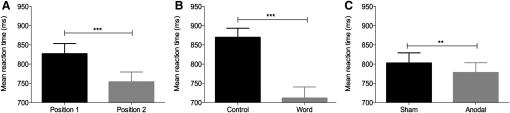
Behavioral Effects of Anodal Transcranial Direct Current Stimulation Main effects of order, i.e., position of run during scanning session (P1 versus P2; A), cue (noise versus word; B), and stimulation (sham versus anodal tDCS [A-tDCS]; C), on naming reaction times (n = 10). Black bars are the control stimuli. Error bars indicate standard error of the mean (SEM). ^∗∗∗^p < 0.001, ^∗∗^p < 0.05.

To directly test this, we targeted left frontal activity using 2 mA A-tDCS during an fMRI study of overt spoken picture naming in ten healthy volunteers (see Figure S1 available online for image quality control). Each of the 107 pictures to be named was presented simultaneously with an auditory cue after a 1000 ms fixation cross. Each picture remained on screen for 2500 ms, and participants were instructed to name the object aloud as quickly and as accurately as possible. To investigate both order and crossmodal repetition BP effects, we presented each picture twice: once with the target's spoken name, and once with an acoustic control cue, a noise-vocoded speech cue (see Supplemental Experimental Procedures for further details). For all subsequent analyses, an effect was considered reliable if it was significant both by subjects (F_1_) and by items (F_2_). For discussion of by-item results only, see the section “Behavioral Data Analyses” in Supplemental Experimental Procedures. As predicted, all participants named the word-cued items significantly faster than the noise-cued (F_1_ and F_2_ p < 0.001; Figure 1), with a mean difference between cue types of 158 ms and accuracy at ceiling for both (Table S2).

On each occasion, the participants completed two 20 min runs of naming. While naming, participants received left frontal A-tDCS (one run real stimulation, the other sham). As expected, there was a robust BP effect of order within a scanning session, with participants naming the items in the second run significantly faster than the first run, akin to a practice effect (F_1_ p < 0.001; F_2_ p < 0.001; Figure 1). Therefore, to determine the effects of A-tDCS, after controlling for order effects, we scanned each participant on two separate occasions at least 5 days apart. The order of real versus sham intervention was counterbalanced across participants and scanning days. Participants reported no adverse sensations during A-tDCS and sham. They could detect a difference between the two conditions (p = 0.07), but they were unable to reliably distinguish which was A-tDCS. This approach permitted measurement of both the behavioral and neural consequences of A-tDCS during (1) real A-tDCS, (2) sham stimulation, and (3) any interactions with the expected BP and NP effects of practice (order) or crossmodal repetition (cues) (see Experimental Procedures for details). No significant differences between the two groups of participants were found in overall naming reaction time (p = 0.68) or any interactions with cue type (p = 0.14), indicating successful counterbalancing of the A-tDCS intervention.

**Figure 2:**
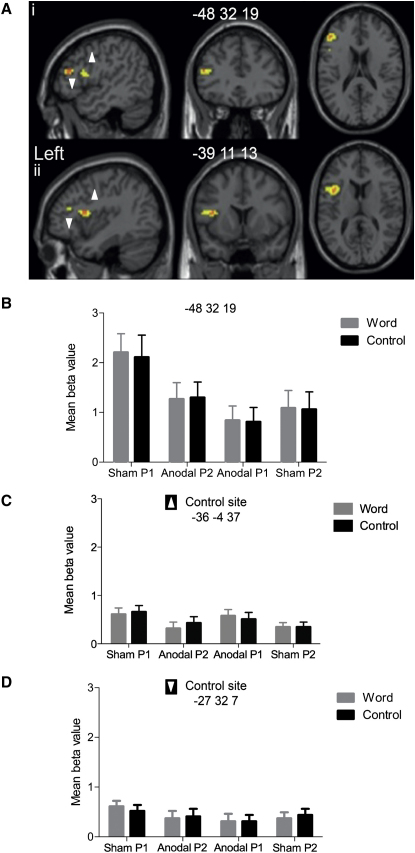
Neural Effects of Anodal Transcranial Direct Current Stimulation (Ai and Aii) Statistical parametric maps showing the greatest reduction in BOLD (orange, peak; yellow, cluster) in inferior frontal sulcus (IFS; z score 4.98; Ai) and ventral premotor cortex (z score 4.62; Aii) as a consequence of A-tDCS. Effects of A-tDCS persist in left IFS peak (z score 4.62) and ventral premotor cortex peak (z score 3.67) when masked exclusively by order effects. (B–D) Mean beta value with SEM of each condition in IFS peak voxel (−48, 32, 19; B) illustrating decreased BOLD response associated with A-tDCS, and in left precentral gyrus (−36, −4, 37; C) and left anterior insula (−27, 32, 7; D) illustrating no effect of A-tDCS. Black bars are the control stimuli. All coordinates are in Montreal Neurological Institute space (x, y, z). See also Figures S1 and S2.

Behaviorally, there was a significant and independent effect of A-tDCS on naming compared to sham. Naming responses remained accurate throughout and were significantly faster in the A-tDCS compared to the sham condition (F_1_ p = 0.02; F_2_ p < 0.001), Figure 1. There were no significant interactions between stimulation, cue, or order effects. Neural effects mirrored behavior (Figure 2B). A-tDCS significantly reduced BOLD signal in left frontal cortex, including Broca's area, compared to sham (p = 0.05 whole-brain analyses). This effect was present for all types of stimuli, irrespective of whether they were cued by words or noise (crossmodal repetition effect), and remained whether they were novel or repeated items within scanning sessions (order effect). Similarly, there was no significant difference between neural effects of A-tDCS across scanning days, indicating that the reduction in BOLD signal associated with A-tDCS could not be explained fully by a repeated task (neural repetition-suppression) effect alone.

**Figure 3:**
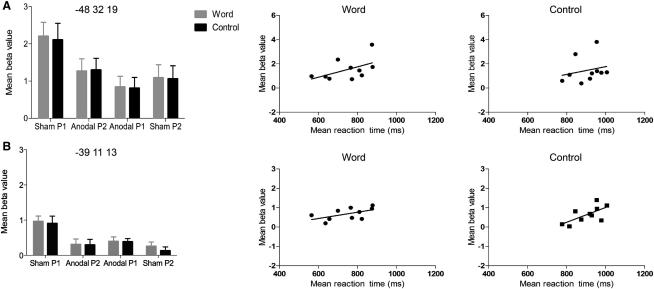
Relationship between Neural and Behavioral Effects Plots of neural effect size and correlations with naming reaction-time data for word- and noise-cued items in inferior frontal sulcus (A) and ventral premotor regions (B) affected by A-tDCS. Left panel reflects mean beta value and SEM for both significant peaks within the vicinity of left Broca's area. Scatter plots at center and right illustrate the spread and relationship between reaction time and activity in each region during sham P1 and anodal P1 conditions. Statistical analyses demonstrated a weak positive correlation in IFS between beta values and reaction time for word cues (r = 0.51, n = 10, p = 0.07, R^2^ = 0.26) but not noise control cues (r = 0.23, n = 10, p = 0.26). These correlations are shown in the top pair of scatter plots (left, word-cued effects; right, noise-cued effects). The bottom pair of scatter plots illustrate a significant correlation in ventral premotor cortex for word (left; r = 0.56, n = 10, p = 0.05, R^2^ = 0.32) and noise control cues (right; r = 0.66, n = 10, p = 0.02, R^2^ = 0.44, all one-tailed). Note the different scales on the y axes of the scatter plots. Anodal P1 and anodal P2 refer to the run order when A-tDCS was delivered within a scanning session. Black bars are the control stimuli.

Importantly, there was a significant positive correlation between A-tDCS behavioral and neural effects. There was a significant weak positive correlation between BOLD response (beta values) and naming reaction time in ventral premotor cortex for both word- (r = 0.56, n = 10, p = 0.05, R^2^ = 0.32) and noise-cued items (r = 0.66, n = 10, p = 0.02, R^2^ = 0.44). A weak positive correlation was approaching significance in the inferior frontal sulcus for word- (r = 0.51, n = 10, p = 0.07, R^2^ = 0.26) but not noise-cued items (r = 0.11, n = 40, p = 0.49, all one-tailed). These data suggest that faster naming responses are associated with a decreased BOLD response in left IFC, including Broca's area (Figure 3).

By contrast, A-tDCS had no detectable impact on neural response in (1) left precentral gyrus and (2) left anterior insula, regions that are also within the vicinity of the anode electrode (Figure S2; Supplemental Experimental Procedures). Exploration of the BOLD response within these regions revealed equivalent activation across both stimulation conditions (sham and A-tDCS). The profiles of the plots from both regions directly contrast with the A-tDCS-modulated effects observed in left ventral premotor cortex and inferior frontal sulcus (Figures 2C and 2D). This indicates (1) a regionally specific rather than global [21] cortical facilitation effect of A-tDCS in left frontal cortex and (2) that left frontal A-tDCS effects are maximal in regions associated with word meaning and retrieval [22] rather than lower-level processes of motor speech output.

Taken together, our data provide direct evidence that (1) left frontal A-tDCS speeds up spoken naming responses; (2) within the stimulated frontal cortex, not all regions are equally affected; (3) Broca's area, but not left precentral or anterior insular cortices, are modulated by A-tDCS; (4) behavioral and neural effects are positively correlated; and (5) effects associated with A-tDCS are independent of well-established crossmodal and order-repetition priming effects. The reduction of BOLD signal in Broca's area may be analogous to the neural priming effects seen in BP experiments. This suggests that A-tDCS concurrent with naming may facilitate responses through a regionally specific neuronal adaptation mechanism in Broca's area. That A-tDCS compared to sham had a facilitatory effect on naming irrespective of cue type (both word- and noise-cued items were faster, with the difference between the two staying the same) highlights that it cannot be associated with the repetition of the phonological form per se (naming associated with the word cue). We propose that left frontal A-tDCS facilitation of naming may be associated with processes of semantic retrieval or the mapping to its associated phonological representation.

Our results support the importance of the left inferior frontal cortex in the normal naming network and point to Broca's area as a candidate site for A-tDCS in rehabilitation protocols aiming to improve anomia in patients whose brain damage spares this region. The presentation of a concurrent task during A-tDCS, as reported here, may be critical to maximally facilitate task-relevant depolarization of membranes so that less synaptic activity is needed to reach a threshold, thereby aiding synaptic modification [23] and resulting in a decreased BOLD response. The novel concurrent combination of tDCS, fMRI, and behavioral measurement as used here may provide unparalleled insight into the neural correlates of tDCS-evoked consequences on behavior and how these regionally specific effects vary with task state.

## Experimental Procedures

### Participants

This study was conducted in the context of a larger research project of speech rehabilitation in aphasic stroke patients, who tend to be older. As such, ten healthy native speakers of English (seven females, three males; mean age 69 years, age range 62–74 years) participated in the study. All participants were left-hemisphere dominant for speech production as determined by a previous fMRI study (R.H., A.P.L., J.C.R., C.J.P., and J.C., unpublished data). Further details of participant selection criteria are reported in Supplemental Experimental Procedures.

### tDCS Stimulation and Concurrent fMRI

A-tDCS stimulation was generated by a specially designed MRI-compatible neuroConn stimulator system (Rogue Resolutions; http://www.rogue-resolutions.com) and delivered at 2 mA continuously for 20 min via a pair of identical MRI-compatible leads and rectangular rubber MRI-compatible electrodes (5 × 7 cm), allowing for a current density of 0.057 mA/cm^2^. For all participants, the anode was placed over the left IFC (equivalent to electrode position FC5 in a 10-10 EEG nomenclature), with the cathode placed over the contralateral frontopolar cortex. Both electrodes and the site on the scalp where the electrodes were to be placed were covered with EEG conductive paste to ensure a flush and comfortable fit between the entire electrode surface and the scalp. Electrodes were secured to the head using 3M Coban elastic wrap bandage. This resulted in a much more efficient blinding process and ensured that participants did not feel pain even with a ramping up of 15 s. The electrodes were placed with an orientation of their connectors to the midline of the head in each participant, in adherence with the manufacturer's MRI safety guidelines. Care was taken with the wire paths: connecting leads were passed backward along the center of the scanner bore, primarily to minimize the possibility of radio frequency-induced heating but also to ensure that any gradient switching-induced AC currents were well below the level that might cause stimulation. The stimulator itself was sited outside the Faraday cage of the scanner, and the stimulating current was fed to the participant through two stages of radio frequency filtration to prevent interference being picked up by the scanner.

A scanner pulse triggered the onset of the stimulation at a given slice in the acquisition sequence. The current was increased slowly during the first 15 s to the desired stimulation threshold (2 mA), termed the “ramp-up” phase, with a further 15 s of stimulation delivered at the thresholded level prior to the onset of the first picture. A constant direct current (2 mA) was delivered for 20 min. At the end of this stimulation period, the current was decreased to 0 mA over 1 s (“ramp-down”). For sham stimulation, the ramp-up phase was followed by 15 s of stimulation prior to the onset of the first picture, which was immediately followed by a 1 s ramp-down phase.

Both stimulation and sham protocols produced sensations of comparable quality (a mild tingling, typically under the electrode placed over the contralateral orbital ridge). Participants habituated to it quickly and reported minimal discomfort with no adverse sensations, phosphenes, or analogous effects during A-tDCS and sham stimulation runs. Participants did detect a difference in sensations between scanning sessions (p = 0.07). However, self-reports indicated that if a difference was detected, participants could not reliably identify which was A-tDCS. The position of the anode and cathode was recorded and reproduced across both scanning sessions.

### Procedure

Half of the participants (n = 5) received a sham stimulation run followed by an A-tDCS run on their first fMRI scanning session. On their second session, the order of intervention was reversed, i.e., they received an A-tDCS run followed by a sham run. The remaining five participants had the opposite order of intervention across scanning sessions. Using this sequencing, the order of intervention was fully counterbalanced across participants. A minimum of 5 and maximum of 7 days separated the two scanning sessions (Table S1 displays the run procedure).

The order of stimuli was pseudorandomized. In the first half of an fMRI run, participants saw each of the 107 pictures paired with either the word or noise cue. Participants then saw the same 107 pictures paired with the remaining cue type in the second half of the run. This procedure was then repeated during the second (P2) run. The order of pictures and accompanying cues was counterbalanced both within and across participants to ensure that the same picture and cue pairing was not presented during the same half of a run on the second scanning session. Visual stimulation was via rear video projection (JVC SX21), and auditory stimulation was via MRI-compatible electrodynamic headphones (Confon). Each picture was preceded by a fixation cross for 1000 ms and displayed for 2500 ms. Auditory cues were presented simultaneously with each picture. Trials were presented in short blocks of six stimuli, separated by a fixation-only rest period of 7 s. The intertrial interval was set to 3920 ms so as to jitter the onset on each trial across acquired brain volumes.

Participants were instructed to name the pictures as quickly and as accurately as possible. They were informed that they would hear either a word or noise cue accompanying each picture, but they were not to wait for this cue to finish before naming the picture. Overt spoken responses were recorded in the scanner using a dual-channel, noise-canceling fiber-optical microphone system (FOMRI III; http://www.optoacoustics.com/). Each response was reviewed offline to verify manual recording of accuracy and used to determine trial-specific reaction times for each participant. Analyses of the reaction-time data only included correct responses (a total of 2.2% trials were excluded because of error) and responses within two standard deviations of the mean for each condition (a total of 3.6% trials excluded). Data were entered into within-subject and between-item 2 × 2 × 2 repeated-measures analyses of variance with cue type (word, noise), stimulation type (sham, A-tDCS), and order (run 1 [P1] or run 2 [P2] within a scanning session) as factors.

## Supplemental Information

Document S1. Two Figures, Two Tables, and Supplemental Experimental Procedures
