# Supplementary material for: Speech Facilitation by Left Inferior Frontal Cortex Stimulation
Source: Curr Biol. 2011 Aug 23;21(16):1403–7. doi: 10.1016/j.cub.2011.07.021 (PMC3315006; doi:10.1016/j.cub.2011.07.021)
Supplement: Document S1. Two Figures, Two Tables, and Supplemental Experimental Procedures [file mmc1.pdf]

Current Biology, Volume *21*

Supplemental Information

Speech Facilitation by Left Inferior Frontal Cortex Stimulation

Rachel Holland, Alex P. Leff, Oliver Josephs, Joseph M. Galea, Mahalekshmi Desikan, Cathy J. Price, John C. Rothwell, and Jennifer Crinion

Inventory of Supplemental Information

Figure S1: Illustrates the effects of A-tDCS on EPI data from a tDCS and fMRI control study illustrating the null effect of the anode electrode on the quality of the EPI images during each functional run. Figure S1 is related to Figure 2A, which shows the greatest reduction in BOLD in two peaks within Broca's area as a consequence of A-tDCS.

Figure S2: Illustrates the A-tDCS peak effects relative to the location of stimulating electrode and control voxels. Figure S2 is related to all data presented in Figure 2, which shows the he greatest reduction in BOLD in two peaks within Broca's area as a consequence of A-tDCS.

Table S1: Details the run procedure.

Table S2: Reports naming response times and accuracy for each condition.

Supplemental Experimental Procedures: Includes further details of participants, stimuli, image acquisition, and analysis procedures and additional by-item analyses of reaction time data.

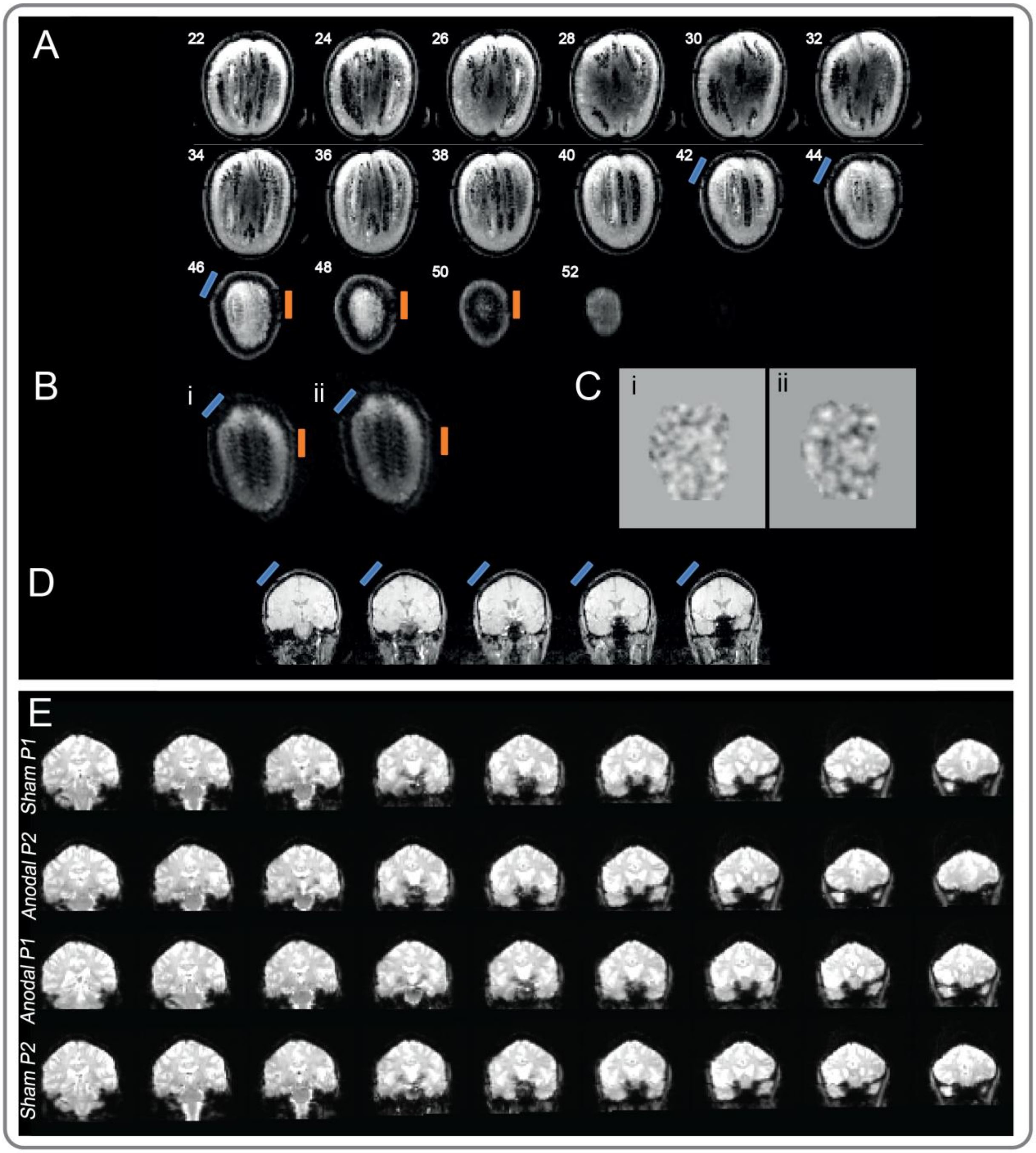
A
22
24
26
28
30
32
34
36
38
40
42
44
46
48
50
52
B
i
ii
C
i
ii
D
E
Sham P1
Anodal P2
Anodal P1
Sham P2

Figure S1.

Figure S1, Related to Figure 2A. Effects of A-tDCS on Echo-Planar Images (EPI)

Field distortions from control (watermelon) and one participant.

(A) Multi-slice coronal view of watermelon field distortion with indices for each slice. Blue bar indicates the location of anodal electrode, orange the cathodal electrode in slices where mild perturbation at the surface under each electrode is evident. Perturbations were localized to the surface layer only of the watermelon. Additional MRI signal arising from the headphones is apparent bilaterally in slices 22-34. These are outside the watermelon and remote from the electrode sites.

(B) EPI from slice 46 (watermelon data) for i) un-stimulated (sham) and ii) stimulated (A-tDCS) runs.

(C) SPM processed T maps of the same watermelon data in coronal view for i) un-stimulated (sham) and ii) stimulated runs. Distribution of "active" voxels is incoherent and no more than expected by chance in both stimulated (A-tDCS) and un-stimulated (sham) runs.

(D) Consistent with the control data, perturbations in the participants' data were also localized to the scalp surface only. Here we illustrate in one participant's coronal sections the effect of the anode electrode on B0 field map data.

(E) EPI data from a single, representative participant in native space illustrating the null effect of the anode electrode on the quality of the EPI images during each functional run. Consistent with data in the top panel (S1 A-D), no signal dropout is apparent in the acquired EPI data due to the presence of the anode electrode. There were no observed perturbations in the brain data for sham and A-tDCS runs. P1 and P2 refer to the run order within a scanning session.

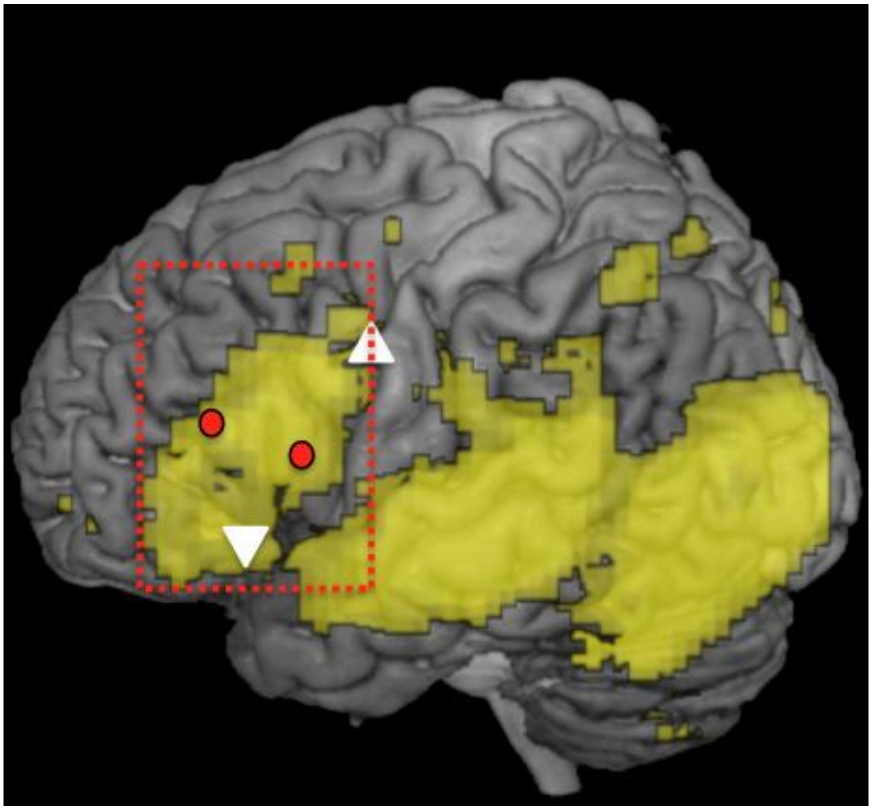

Figure S2, Related to Figure 2. Regional Specificity of the A-tDCS Effect

Regional-specific A-tDCS peak effects are illustrated relative to the location of stimulating electrode. Loci are rendered onto a cortical surface statistical parametric map of the main effect of naming > rest irrespective of stimulation condition, whole-brain analyses ($p = 0.05$ corrected). Vertical white triangle = the left precentral gyrus peak (-36 -4 37) activated in the normal naming network. Inverted white triangle = control voxel equidistant (-27 32 7) to peak voxels modulated by tDCS (c.f. Figure 2C and D for plot of effect sizes from the left precentral gyrus and control voxels respectively). Red dots = the peak voxels modulated by A-tDCS (inferior frontal sulcus, -48, 32, 19 and ventral premotor, -39 11 13). Red dashed line indicates the approximate edge of the electrode ($35cm^2$) placed over left frontal cortex. Note the proximal location of the left precentral gyrus is closer to the electrode tip and delivery point of stimulation than peaks within Broca's area (c.f. Figure 2C for plot of effect size). All coordinates are in MNI space (x,y,z). See Supplemental Experimental Procedures for further details of selection of the left precentral gyrus and control voxels.

Table S1. Timeline of the Experimental Run Procedure

		Run 1 (P1)		Run 2 (P2)
**n = 5**	Scanning Session 1	Sham	→	A-tDCS
	Scanning Session 2	A-tDCS	→	Sham
**n = 5**	Scanning Session 1	A-tDCS	→	Sham
	Scanning Session 2	Sham	→	A- tDCS

Graphically displays the two, counterbalanced orders of intervention used. P1 and P2 refer to the run order within a scanning session.

Table S2. Naming Performance

	Reaction Time (ms)		Accuracy			
	Mean		Mean		Min and Max	
	Word	Noise	Word	Noise	Word	Noise
**Sham P1**	767.4 (40)	913.4 (27.3)	1.00 (0.03)	0.98 (0.05)	0.98, 1.00	0.95, 1.00
**Anodal P2**	662.6 (29)	823 (29.8)	1.00 (0.02)	0.98 (0.08)	0.98, 1.00	0.93, 1.00
**Anodal P1**	728.6 (35.3)	897 (35.3)	0.99 (0.03)	0.96 (0.07)	0.98, 1.00	0.92, 0.99
**Sham P2**	686 (28.6)	842.7 (26.9)	1.00 (0)	0.98 (0.08)	1.00, 1.00	0.93, 1.00

Mean reaction times and accuracy for each experimental condition and cue type. Standard error of mean (SEM) in parentheses. (ms) = milliseconds; Accuracy = proportion correct and minimum (Min) and maximum (Max) values. P1 and P2 refer to the run order within a scanning session, see Table S1.

Supplemental Experimental Procedures

Participants
Participants were selected based on previous behavioral performance during a speech-priming fMRI task. The main criterion for selection was a word versus control cue priming effect of greater than 85 ms. All had normal hearing and no previous history of implants, neurological or psychiatric disease. All participants gave their written and informed consent to participate in the study.

Stimuli
The stimulus set consisted of 107 black and white line drawings of objects. All object names were monosyllabic and CVC in terms of phonological structure. CVC structure refers to the constituent phoneme, rather than grapheme sequence of a word. For example, pig and star are both CVC in terms of phonological structure, despite star beginning with two consonants /st/. Using the norms from the International Picture Naming Project (IPNP) [1] all selected objects had a name agreement (i.e., percentage of IPNP participants producing the target name) of greater than 75%.

Auditory cues were either (i) the target object name (i.e., word cue) or (ii) an unintelligible acoustic control cue (noise cue). To create the auditory cues, the target name for each object was digitally recorded (sampling rate 44.1kHz) from a male native speaker of English in a soundproof room. To generate the acoustic control cues, each of the spoken word cues were spectrally rotated [2] and then submitted to a noise-vocoding routine [3] using a single level of filter band noise vocoding. This procedure leaves the temporal envelope of the spoken token unaltered and preserves the spectro-temporal complexity whilst rendering the auditory signal unintelligible by inverting the frequency spectrum. This auditory condition controls for auditory alerting effects and acoustic load and has successfully been used as a control condition in previous neuroimaging studies [4-6]

Imaging
Whole-brain imaging was performed on a 3T Siemens TIM-Trio system (Siemens, Erlangen, Germany) at the Wellcome Trust Centre for Neuroimaging. Using a 12-channel head coil we acquired T2*-weighted echo-planar images (EPI) with BOLD contrast. Each EPI comprised 48 AC/PC-aligned axial slices with sequential ascending acquisition; slice thickness of 2 mm, 1 mm inter-slice gap and a 3 x 3 mm in-plane resolution. Volumes were acquired with a repetition time (TR) of 3,360 ms per volume and the first six volumes of each session were discarded to allow for T1 equilibration effects. A total of 350 volume images (344 volumes of interest, 6 dummy scans) were acquired in two consecutive runs within each session; each lasting approximately 20 minutes. Prior to the first functional run of each scanning session, a gradient field map was acquired for each participant for later B0 field distortion correction of functional images. The same scanner and hardware were used for the acquisition of all images.

The functional data were preprocessed using Statistical Parametric Mapping software (SPM8; www.fil.ion.ucl.ac.uk/spm) running under Matlab 7.7 (MathWorks, Natick, MA). The first six volumes were discarded and all subsequent volumes from each participant were realigned and unwarped, using the first image as reference and resliced with sinc interpolation. The functional images were then spatially normalized to the standard T2* template within SPM normalization software. Functional data were spatially smoothed, with a 8mm full-width at half-

maximum isotropic Gaussian kernel to allow for residual variability after spatial normalization and to permit application of Gaussian random field theory for corrected statistical inference.

Statistical analyses were first performed in a subject-specific fashion. To remove low-frequency drifts, the data were high-pass filtered using a set of discrete cosine functions with a cut-off period of 128 sec. Each condition and cue type was modelled separately as an event by convolving it with the SPM canonical haemodynamic response function (HRF). We used presentation of the pictorial and auditory cue as the onset of the event to model the naming response. Movement realignment parameters were included as covariates of no interest. The resulting stimulus-specific parameter estimates were calculated for all brain voxels using the General Linear Model. Contrast images were computed for each cue type relative to rest for whole brain analyses at the second level.

Regional Specificity of the A-tDCS Effects, Related to Figure S3

Two additional voxels within the left frontal cortex, within the vicinity of the anode electrode and within the naming network were investigated. The first, located at the peak response ($p = 0.05$, corrected) within the left precentral gyrus (-36 -4 37; z-score 6.24), was identified by masking the main effect of naming > rest irrespective of stimulation condition, whole brain analyses ($p = 0.05$ corrected), by an anatomical mask of Brodmann Area 6 [7] from the WFU Pickatlas [8]. The second, located in left anterior insula gyrus (-27 32 7), was identified as an additional control voxel that was located at a distance equal to the Euclidean distance between to the two fMRI voxel peaks reported for the main effect of tDCS. The neural responses within these regions were then plotted for the contrast of interest.

Behavioral Data Analyses

Statistical analyses were performed by-subjects and by-items. The main effect of stimulation (sham, A-tDCS), order (P1, P2) and cue type were significant both by-subjects (F_1) and by-items (F_2). By-subject analyses revealed no significant interactions between factors. However, the by-item analysis revealed a significant interaction between stimulation type (sham, A-tDCS) and cue type (word, noise) with faster naming responses during anodal stimulation of picture naming when paired with word cues ($F_2(1,106)=8.33$, $p=0.005$). A further three-way interaction between stimulation, order and cue type was approaching significance ($F_2(1,106)=3.703$, $p=0.06$). No other interactions were approaching significance.

tDCS and fMRI Image Quality and Safety Control Study

Prior to scanning the human participants we conducted a control anodal tDCS (A-tDCS) concurrent with fMRI study. The purpose of the control study was twofold: (i) to ensure the safety of concurrent tDCS and fMRI and (ii) to quantify any noise effects in the images induced by A-tDCS delivered simultaneously with the task stimuli. Here we delivered 2 mA stimulation for 20 minutes concurrently with the identical stimulus delivery set-up as used in the main study to an inert object (a watermelon). A watermelon of similar size to a human head was chosen as it has different tissue densities (outer skin, inner rim, water filled and fleshy tissue) and a continuous 2 mA anodal DC could be passed through the surface. The headphones and electrodes were positioned on the object in the same orientation, and with the same procedure as was used for the reported main study. Additionally, an MRI compatible fiber-optic thermometer probe (Opsens, Canada) was placed under the stimulating electrodes. Results indicated that (i) during stimulation no significant changes in surface temperature were detected over time; and

(ii) in distortion correction field maps only minimal perturbation of signal was observed at the electrode site. This was restricted to the surface of the watermelon only (c.f. Figure S1A).

Preprocessing of the acquired functional data was equivalent to participant data (except for the omission of the spatial normalization step) and the data were modeled at the first-level in the same manner to individual subject data and equivalent contrasts were computed. At the corrected level of statistical thresholding, there were no effects of A-tDCS on sham or stimulated runs. The analysis was repeated at a lenient threshold (peak: P = 0.001, uncorrected; extent 10 contiguous voxels,) and again there were no significant clusters. Furthermore, the numbers of voxels "active" overall were no more than expected by chance and the distribution of "active" voxels across the whole object was not localized to the vicinity of the electrodes (Figure S1B i and ii). These results indicate that the specific effects of A-tDCS reported in the naming study cannot be due to imaging artifacts induced by concurrent stimulation.

Supplemental References

1. Szekely, A., Jacobsen, T., D'Amico, S., Devescovi, A., Andonova, E., Herron, D., Lu, C.C., Pechmann, T., Pleh, C., Wicha, N., Federmeier, K., Gerdjikova, I., Gutierrez, G., Hung, D., Hsu, J., Iyer, G., Kohnert, K., Mehotcheva, T., Orozco-Figueroa, A., Tzeng, A., Tzeng, O., Arevalo, A., Vargha, A., Butler, A.C., Buffington, R., and Bates, E., (2004). A new on-line resource for psycholinguistic studies. J. Mem. Lang. *51*, 247-250.
2. Blesser, B., (1972). Speech perception under conditions of specteal transformation: 1 Phonetic characteristics. J. Speech Hear. Res. *15*, 5-41.
3. Shannon, R.V., Zeng, F.G., Kamath, V., Wygonski, J., and Ekelid, M., (1995). Speech recognition with primarily temporal cues. Science. *270*, 303-304.
4. Obleser, J., Wise, R.J.S., Dresner, M.A., and Scott, S.K., (2007). Functional integration across brain regions improves speech perception under adverse listening conditions. J. Neurosci. *27*, 2283-2289.
5. Narain, C., Scott, S.K., Wise, R.J.S., Rosen, S., Leff, A., Iversen, S.D., and Matthews, P.M., (2003). Defining a left-lateralized response specific to intelligible speech using fMRI. Cereb. Cortex. *13*, 1362-1368.
6. Scott, S.K., Blank, C.C., Rosen, S., and Wise, R.J.S., (2000). Identification of a pathway for intelligible speech in the left temporal lobe. Brain. *123*, 2400-2406.
7. Lancaster, J.L., Woldorff, M.G., Parsons, L.M., Liotti, M., Freitas, C.S., Rainey, L., Kochunov, P.V., Nickerson, D., Mikiten, S.A., and Fox, P.T., (2000). Automated Talairach atlas labels for functional brain mapping. Hum. Brain Mapp. *10*, 120-131.
8. Maldjian, J.A., Laurienti, P.J., Kraft, R.A., and Burdette, J.H., (2003). An automated method for neuroanatomic and cytoarchitectonic atlas-based interrogation of fMRI data sets. Neuroimage. *19*, 1233-1239.
